# RESPIRE Score: Derivation and Validation of a New Risk Score for Prediction of Community-acquired Pneumonia Caused by Resistant Pathogens

**DOI:** 10.1093/ofid/ofag319

**Published:** 2026-05-21

**Authors:** Lorenzo Pelagatti, Martina De Marco, Emma Bosco, Tullio Catalucci, Michele Gilardoni, Francesca Mangani, Michele Spinicci, Alessandro Bartoloni, Gian Maria Rossolini, Peiman Nazerian, Simone Vanni

**Affiliations:** Department of Experimental and Clinical Medicine, University of Florence, Florence, Italy; Emergency Department, Careggi University Hospital, Florence, Italy; Department of Experimental and Clinical Medicine, University of Florence, Florence, Italy; Department of Experimental and Clinical Medicine, University of Florence, Florence, Italy; Department of Experimental and Clinical Medicine, University of Florence, Florence, Italy; Department of Experimental and Clinical Medicine, University of Florence, Florence, Italy; Department of Experimental and Clinical Medicine, University of Florence, Florence, Italy; Microbiology and Virology Unit, Careggi University Hospital, Florence, Italy; Department of Experimental and Clinical Medicine, University of Florence, Florence, Italy; Infectious and Tropical Diseases Unit, Careggi University Hospital, Florence, Italy; Department of Experimental and Clinical Medicine, University of Florence, Florence, Italy; Infectious and Tropical Diseases Unit, Careggi University Hospital, Florence, Italy; Department of Experimental and Clinical Medicine, University of Florence, Florence, Italy; Microbiology and Virology Unit, Careggi University Hospital, Florence, Italy; Department of Experimental and Clinical Medicine, University of Florence, Florence, Italy; Emergency Department, Careggi University Hospital, Florence, Italy; Department of Experimental and Clinical Medicine, University of Florence, Florence, Italy; High Dependency Unit, Careggi University Hospital, Florence, Italy

**Keywords:** antibiotic therapy, clinical score, community-acquired pneumonia, multidrug-resistant pathogens

## Abstract

**Background:**

Community-acquired pneumonia (CAP) remains a leading cause of infectious mortality worldwide. The increasing prevalence of multidrug-resistant (MDR) pathogens challenges the established empirical antibiotic therapy schemes. This study aimed to derive and validate a clinical score (RESPIRE) to identify CAP-MDR patients.

**Methods:**

We conducted an observational study in a setting of medium-high multidrug-resistant (MDR) endemicity. The primary outcome was the derivation of a clinical score predicting MDR-CAP. A retrospective derivation cohort (January 2022–December 2023) including adult patients hospitalized with microbiologically confirmed CAP by culture on respiratory samples was used for score derivation. A prospective cohort (January 2024–December 2025) including adult patients with the same clinical features was used for score validation.

**Results:**

The derivation cohort included 275 patients (mean age 68.3 years), with an MDR prevalence of 30.1% (n = 83). The RESPIRE score assigns 1.5 points for recent hospitalization and prior antibiotic use, and 1 point each for enteral feeding, poor functional status, and residence in long-term care facilities (maximum 6 points). A cutoff ≥ 2 showed optimal performance in predicting CAP-MDR (AUROC 0.85; 95% CI 0.80–0.91), outperforming other scores except DRIP. Validation in 141 patients (39% MDR) confirmed an AUROC of 0.88 (95% CI 0.74–0.89) showing better performance compared to other diagnostic models.

**Conclusions:**

The RESPIRE score outperformed HCAP criteria and other clinical scores in predicting CAP-MDR. Its implementation in protocols could support antimicrobial stewardship for empiric treatment of cases of CAP presenting at the Emergency Department. A multicenter validation is warranted to confirm the performance of the RESPIRE score.

**Trial registration number:**

NCT07425561

Community-acquired pneumonia (CAP) is among the leading causes of death worldwide and represents the foremost cause of mortality from infectious diseases, accounting for more than 2.4 million deaths annually [1–3]. Early initiation of empirical antibiotic therapy is associated with improved survival outcomes [4, 5]. In hospitalized patients with CAP, standard empirical treatment has historically consisted of a β-lactam, namely, amoxicillin or a cephalosporin, combined with a macrolide or tetracycline, or monotherapy with a fluoroquinolone [6–8].

However, the increasing prevalence of CAP caused by multidrug-resistant (MDR) pathogens (CAP-MDR) has complicated empirical antibiotic selection [9, 10]. MDR pathogens are defined as organisms nonsusceptible to at least 1 agent in 3 or more antimicrobial classes [11]. The most frequently implicated MDR pathogens in CAP include methicillin-resistant *Staphylococcus aureus* (MRSA) and MDR Gram-negative bacilli (eg, *Pseudomonas aeruginosa*, *Klebsiella pneumoniae* and other *Enterobacterales*, and *Acinetobacter baumannii*) [12].

Early identification of infections caused by MDR pathogens is essential, as standard empirical regimens may be ineffective and broader-spectrum coverage may be required.

In 2005, the American Thoracic Society and the Infectious Diseases Society of America (ATS/IDSA) introduced the category of healthcare-associated pneumonia (HCAP) to identify patients at increased risk of MDR infection [6]. However, HCAP clinical criteria were subsequently shown to have suboptimal sensitivity and specificity for predicting CAP-MDR [13], leading to unnecessary overuse of broad-spectrum antibiotics [14–17]. Accordingly, the 2019 ATS/IDSA guidelines no longer recommend using the HCAP classification to guide empirical therapy. Instead, they advise considering locally validated risk factors for MDR pathogens—particularly MRSA and *P. aeruginosa*—and the use of clinical prediction scores [8]. This underscores the need for locally validated risk stratification tools to identify CAP patients at risk for MDR infection.

Several prediction scores have been developed in recent years based on cohorts from North America and Asia [18–22], whereas only 2 studies have been conducted in Europe [23, 24].

The aim of the present study was to derive a clinical prediction score for MDR infection in CAP using an Italian cohort and to validate the model prospectively in an independent patient cohort.

## METHODS

### Study Design and Setting

This was a single-center, nonprofit observational study consisting of a retrospective derivation phase followed by a prospective validation phase. Patients were enrolled during 2 consecutive, nonoverlapping periods: 1 January 2022–31 December 2023 (retrospective cohort) and 1 January 2024–31 December 2025 (prospective cohort).

The study was conducted at the Emergency Department (ED) and Emergency Sub-intensive Care Unit of a Careggi University Hospital, Florence, Tuscany, Italy, in collaboration with the Units of Microbiology and Virology and of Infectious and Tropical Diseases. Careggi is a large tertiary academic hospital serving approximately 650 000 inhabitants locally and acting as referral center for a population of approximately 1.6 million.

Recent regional surveillance data highlight the substantial burden of antimicrobial resistance in Tuscany [25].

### Retrospective Derivation Cohort

During the 2-year retrospective phase, data were collected to establish a derivation cohort of patients with microbiologically confirmed bacterial CAP presenting at the Emergency Department (ED) or at another hospital ward. For the initial screening, microbiological test requests were reviewed to identify patients who underwent respiratory sample testing for etiologic diagnosis within 48 hours of hospital presentation.

Microbiological test requests recorded in the laboratory information system were initially screened to identify patients who underwent diagnostic testing on respiratory samples (considered the gold standard for etiologic diagnosis). Electronic medical records were subsequently reviewed by the investigators to construct the final study database.

Only patients with culture-confirmed bacterial pneumonia and available antimicrobial susceptibility testing were included. Patients with pneumonia caused by MDR organisms constituted the study group, whereas those with pneumonia caused by non-MDR bacteria served as controls. MDR-CAP was defined as isolation of bacterial pathogens nonsusceptible to at least 1 agent in 3 or more antimicrobial classes potentially active against those pathogens [11]. MDR pathogens are clinically relevant as they are more likely to be inadequately covered by standard empirical antibiotic regimens (eg, including β-lactam/macrolide combinations or fluoroquinolone monotherapy).

To minimize selection bias, only patients with microbiologically confirmed pneumonia and positive respiratory cultures were included. Patients with negative respiratory cultures (reported in 40%–60% of cases in the literature) were excluded [26], as MDR etiology could not be reliably ruled out in culture-negative cases. Accordingly, the score was derived to predict MDR bacterial CAP in a microbiologically adjudicated population, rather than in all consecutive patients presenting with suspected CAP.

Cases with a positive molecular syndromic panel for bacterial pathogens but negative respiratory cultures—regardless of the detection of resistance genes—were excluded. The rationale for this choice was methodological: culture results with antimicrobial susceptibility testing represent the reference standard for defining MDR phenotypes, whereas molecular detection alone may identify colonization, low bacterial load, or resistance genes whose phenotypic expression cannot be reliably confirmed. For this reason, inclusion of molecular-only positive cases could have introduced misclassification of the outcome.

Given the potential for false-negative cultures due to antibiotic exposure at the time of respiratory sample collection, patients with negative microbiological results cannot be reliably considered true negatives and were therefore excluded from the primary analysis. In an exploratory sensitivity analysis, we conducted a conservative worst-case scenario by classifying all culture-negative patients as non-MDR (Supplementary material).

### Prospective Validation Cohort

During the subsequent 2-year prospective phase, data were collected to establish a validation cohort of ED patients with pneumonia. The performance of the clinical prediction score derived from the retrospective cohort was assessed in a purely observational manner.

The validation cohort included patients diagnosed with pneumonia in the ED who underwent microbiological testing on respiratory samples at the discretion of the treating physician. No additional diagnostic procedures were mandated by the study protocol. After initial screening, only patients with positive respiratory cultures were included, for the same methodological reasons described above.

The Departments of Emergency Medicine were responsible for patient assessment, enrollment, respiratory sample collection, and clinical data acquisition. The Department of Microbiology and Virology was responsible for identifying eligible microbiological test requests and extracting microbiological data during the study period.

### Study Objectives

The primary objective of the study was to derive a clinical prediction score for CAP-MDR in patients presenting to the ED or hospital from the community with a diagnosis of pneumonia (RESPIRE score: Risk Evaluation Score for Pneumonia Involving Resistant Entities).

Secondary objectives were: (1) to prospectively validate the derived clinical score in an independent patient cohort; (2) to compare the predictive performance of the newly developed score with existing validated tools, including the DRIP, Shorr, Park, Shindo, Aliberti, and Schreiber scores, as well as the ATS/IDSA HCAP criteria [4, 6, 18–23]; (3) to assess positive predictive value (PPV), negative predictive value (NPV), sensitivity, specificity, positive and negative likelihood ratios (LR+ and LR−), and the optimal diagnostic cutoff; (4) to evaluate model calibration in both the derivation and validation cohorts; (5) to assess clinical utility using decision curve analysis (DCA).

### Inclusion and Exclusion Criteria

All patients aged >18 years who underwent respiratory sample collection within 48 hours from hospital presentation and had positive respiratory cultures with microbiologically confirmed diagnosis of bacterial CAP were eligible for inclusion.

Pneumonia was clinically defined as the presence of at least 2 of the following signs or symptoms: body temperature <36.0°C or >38.0°C; respiratory rate >20 breaths/min; oxygen saturation on room air <90%; arterial partial pressure of oxygen (PaO_2_) < 60 mmHg; cough; sputum production; white blood cell count <4000/μL or >10 000/μL; or band forms >10%, together with radiographic evidence of a new pulmonary infiltrate or cavitation.

During the prospective phase, only patients whose microbiological samples were collected in the Emergency Department (emergency room, high dependency unit, or emergency medicine ward) were enrolled.

Exclusion criteria were lack of informed consent; age <18 years or >90 years; pregnancy; hospital presentation >48 hours after admission (hospital-acquired pneumonia, HAP); viral or fungal pneumonia; and negative respiratory cultures.

### Diagnostic Testing for Pathogen Identification

Details of microbiological diagnostic procedures are provided in the Supplementary material (Materials and Methods section).

### Variables

Clinical and anamnestic data were collected, including vital signs, laboratory parameters, radiological and ultrasound findings, microbiological results, and clinical outcomes (mortality, length of hospital stay, and readmission).

### Statistical Analysis

Univariate analysis of categorical variables was performed using the χ^2^ test or Fisher's exact test, as appropriate. Continuous variables were analyzed using the independent-samples *t* test or the Mann–Whitney *U* test for non-normally distributed data. A 2-sided *P* value <.05 was considered statistically significant.

Missing data were <5% for all candidate predictors; therefore, complete-case analysis was performed without imputation.

The association between risk factors for MDR bacterial pneumonia and non-MDR pneumonia was assessed using grouped data analysis and logistic regression. Ninety-five percent confidence intervals (95% CI) for proportions and differences between proportions were calculated using the Z test and the modified Wilson method, respectively.

Multivariable logistic regression models were constructed to identify independent risk factors for MDR pneumonia. Candidate predictors were selected *a priori* based on variables consistently associated with MDR pneumonia in the literature. Variables with *P* < .20 in univariate analysis were entered into the initial multivariable model and subsequently evaluated for their contribution to the final model.

A clinical prediction score (RESPIRE) was derived through sequential inclusion of significant predictors and evaluation of diagnostic performance (sensitivity, specificity, positive predictive value [PPV], negative predictive value [NPV], positive and negative likelihood ratios [LR+ and LR−], and area under the receiver operating characteristic curve [AUROC]). To construct the RESPIRE score, regression coefficients from the final multivariable model were rescaled by dividing each beta coefficient by the smallest absolute beta value and rounding to the nearest 0.5, ensuring proportional weighting while preserving clinical applicability. AUROC values were calculated using standard methods, and differences between AUROCs were assessed using the Z test and DeLong's test.

Model calibration was evaluated in both the derivation and validation cohorts using the Hosmer–Lemeshow goodness-of-fit test and calibration plots. Patients were stratified into quintiles of predicted risk based on ranked predicted probabilities to account for the discrete nature of the score.

To reduce the risk of overfitting during derivation of the RESPIRE score, predictor selection was performed using penalized logistic regression with least absolute shrinkage and selection operator (LASSO), with the penalty parameter determined by 10-fold cross-validation. This was followed by an iterative selection procedure and internal validation using bootstrap resampling with replacement to assess predictor stability and estimate residual optimism in model performance.

The DRIP, Shorr, Niederman, Park, Shindo, Aliberti, and Schreiber scores [18–23], as well as the ATS/IDSA HCAP criteria [6], were applied to the derivation cohort, and their diagnostic performance was compared with that of the RESPIRE score. The same analyses were subsequently performed in the validation cohort.

Clinical utility was assessed using decision curve analysis, estimating the net benefit of the RESPIRE score compared with “treat-all” and “treat-none” strategies across a wide range of threshold probabilities (validation cohort). To assess clinical applicability, we performed an additional analysis estimating the potential impact of the RESPIRE score on empirical antibiotic prescribing. Based on the proposed cutoff, patients were classified as candidates or not for broad-spectrum coverage, and the number of potentially undertreated and overtreated patients was calculated and compared with HCAP-based criteria.

Unless otherwise specified, all statistical analyses were performed using SPSS Statistics for Windows, version 22.0 (IBM Corp., Armonk, NY, USA); graphical representations were generated using SPSS and Jamovi (The Jamovi Project).

### Ethical Approval

The study “RESPIRE: Derivation and Validation of the Risk Evaluation Score for Pneumonia Involving Resistant Entities” received approval from the Ethics Committee (CEAVC No. 29707) (ClinicalTrials.gov: NCT07425561).

## RESULTS

### Derivation Cohort

Between 1 January 2022 and 31 December 2023, the derivation cohort included 275 patients (Supplementary Figure 1, study flowchart), with a mean age of 68.3 ± 16.6 years. The prevalence of multidrug-resistant (MDR) infections was 30.1% (n = 83) (Table 1).

**Table 1.** Comorbidities of Patients in the Derivation and Validation Cohorts

Details	Derivation Cohort				Validation Cohort			
	Population (n = 275)	MDR (N = 83)	Non-MDR (N = 192)	*P* Value	Population (N = 141)	MDR (N = 55)	Non-MDR (N = 86)	*P* Value
Age (y)	68.3 ± 16.6	71.6 ± 14.6	66.8 ± 17.2	0.17	72.3 ± 15.6	73.4 ± 12.6	70.1 ± 14.4	.6
Female sex	103/275 (37.4%)	23/83 (27.7%)	80/192 (41.6%)	0.07	59/141 (41.8%)	19/55 (34.5%)	40/86 (46.5%)	.16
Comorbidity	Derivation Cohort				Validation Cohort			
Arterial hypertension	137/275 (49.8%)	46/83 (55.4%)	91/192 (47.4%)	0.24	76/141 (53.9%)	33/55 (60%)	43/86 (50%)	.3
COPD	76/275 (27.6%)	28/83 (33.7%)	48/192 (25%)	0.14	47/141 (33.3%)	20/55 (36.4%)	27/86 (31.4%)	.59
T2DM	46/269 (16.7%)	22/82 (26.5%)	24/192 (12.5%)	0.01	32/141 (22.7%)	17/55 (30.9%)	15/86 (17.4%)	.07
Asthma	17/275 (6.2%)	2/83 (2.4%)	15/192 (7.8%)	0.11	6/141 (4.3%)	1/55 (1.8%)	5/86 (5.8%)	.4
Bronchiectasis	32/275 (11.6%)	7/83 (8.4%)	25/192 (13%)	0.32	14/141 (9.9%)	5/55 (9.1%)	9/86 (10.5%)	1
CHF	43/275 (15.6%)	18/82 (21.7%)	25/192 (13%)	0.07	19/141 (13.5%)	10/55 (18.2%)	9/86 (10.5%)	.21
CAD	39/273 (14.3%)	15/83 (18.1%)	24/190 (12.6%)	0.26	22/140 (15.7%)	10/55 (18.2%)	12/85 (14.1%)	.64
AF	41/275 (14.9%)	22/83 (26.5%)	19/190 (9.9%)	0.001	23/141 (16.3%)	14/55 (25.5%)	9/86 (10.5%)	.03
DVT/PE	26/275 (9.5%)	9/83 (10.8%)	17/192 (8.9%)	0.66	14/141 (9.9%)	7/55 (12.7%)	7/86 (8.1%)	.4
Stroke	27/274 (9.9%)	14/83 (16.9%)	13/191 (6.8%)	0.01	16/141 (11.3%)	11/55 (20%)	5/86 (5.8%)	.01
TIA	4/275 (1.5%)	2/83 (2.4%)	2/192 (1%)	0.59	2/141 (1.4%)	2/55 (3.6%)	0/86 (0%)	.15
CKD	44/275 (16%)	17/83 (20.5%)	27/192 (14.1%)	0.21	23/141 (16.3%)	11/55 (20%)	12/86 (14%)	.36
Cirrhosis/liver disease	9/275 (3.3%)	4/83 (4.8%)	5/192 (2.6%)	0.52	6/141 (4.3%)	3/55 (5.5%)	3/86 (3.5%)	.68
Immunosuppression	47/275 (17.1%)	15/83 (18.1%)	32/192 (16.7%)	0.86	30/141 (21.3%)	8/55 (14.5%)	22/86 (25.6%)	.14
Solid organ and/or stem cell transplant	5/274 (1.8%)	3/82 (3.7%)	2/192 (1%)	0.16	3/140 (2.1%)	1/54 (1.9%)	2/86 (2.3%)	1
Cognitive impairment	50/275 (18.2%)	28/83 (33.7%)	22/192 (11.5%)	<0.001	44/141 (31.2%)	26/55 (47.3%)	18/86 (20.9%)	.001
Active cancer	45/274 (16.4%)	12/83 (14.5%)	33/191 (17.3%)	0.60	34/141 (24.1%)	12/55 (21.8%)	22/86 (25.6%)	.69
Cancer in negative follow-up	38/275 (13.8%)	7/83 (8.4%)	31/192 (16.2%)	0.13	23/141 (16.3%)	5/55 (9.1%)	18/86 (20.9%)	.1

Abbreviations: COPD, chronic obstructive pulmonary disease; T2DM, type 2 diabetes mellitus; CHF, chronic heart failure; CAD, coronary artery disease; AF, atrial fibrillation; DVT, deep vein thrombosis; PE, pulmonary embolism; TIA, transient ischemic attack; CKD, chronic kidney disease.

Regarding clinical outcomes, 244/275 patients (88.7%) were hospitalized following Emergency Department presentation; 17/275 (6.2%) were discharged home; 10/275 (3.6%) were discharged to a healthcare facility; and 4/275 (1.5%) died in the Emergency Department. The overall 30-day mortality rate was 28.7% (79/275).

Differences in comorbidity distribution are reported in Table 1. Vital signs, arterial blood gas parameters, and laboratory findings for both the derivation and validation cohorts are reported in Supplementary Table 1 (Supplementary material).

In both the derivation and validation cohorts, most respiratory samples consisted of bronchoalveolar lavage (BAL) specimens (>85%) (Table 2). The most frequently identified pathogens are reported in Table 2.

**Table 2:** Microbiological Investigation Results for the Derivation and Validation Cohorts

	Derivation Cohort (n = 275) No. Patients % Patients		Validation Cohort (n = 141) No. Patients % Patients	
Sample				
ETA	8	2.9%	7	5%
BAS	33	12%	6	4.2%
BAL	234	85.1%	128	90.8%

Microbiological investigation	No patients (positive)	% patients (% positive)	No patients (positive)	% patients (% positive)
Culture on respiratory samples	275 (275)	100% (100%)	141 (141)	100% (100%)
Syndromic molecular panel on respiratory samples	252 (242)	91.6% (88.0%)	140 (138)	99.3% (97.9%)
Urinary antigens	148 (19)	54.8% (6.9%)	99 (11)	70.7% (7.8%)
Blood cultures	125 (30)	45.8% (10.9%)	67 (15)	47.5% (10.6%)

Bacteria	N = 275 (MDR = 83)^c^	% total	N = 141 (MDR = 55)^c^	% total
Gram+				
*Streptococcus pneumoniae*	21 (2)	7.6%	7 (1)	5.0%
* MSSA*	60 (5)	21.8%	26 (2)	18.4%
* MRSA*	4 (4)	1.5%	3 (3)	2.1%
* Group A beta-hemolytic streptococcus*	6 (0)	2.2%	0 (0)	0.0%
* Group B beta-hemolytic streptococcus*	3 (0)	1.1%	0 (0)	0.0%
*Streptococcus anginosus* group	1 (0)	0.4%	1 (0)	0.7%
*Corynebacterium striatum*	7 (6)	2.5%	2 (2)	1.4%
Other Gram+	2^a^ (0)	0.7%	2^b^ (0)	1.4%
Gram−				
*P. aeruginosa*	38 (12)	13.8%	21 (6)	14.9%
*Escherichia coli*	34 (10)	12.4%	17 (5)	12.1%
*K. pneumoniae*	61 (25)	22.2%	35 (26)	24.8%
*Klebsiella oxytoca*	10 (0)	3.6%	3 (1)	2.1%
Other *Klebsiella* species	3 (1)	1.1%	1 (0)	0.7%
*Haemophilus influenzae*	44 (0)	16%	14 (0)	9.9%
*Enterobacter cloacae*	7 (4)	2.5%	1 (1)	0.7%
Other *Enterobacteriaceae*	3 (2)	1.1%	1 (0)	0.7%
*A. baumannii* complex	13 (11)	4.7%	10 (10)	7.1%
Other *Acinetobacter* species	2 (0)	0.7%	2 (0)	1.4%
*Proteus mirabilis*	11 (4)	4.0%	7 (3)	5.0%
*Moraxella catarrhalis*	8 (0)	2.9%	3 (0)	2.1%
*Stenotrophomonas maltophilia*	6 (2)	2.2%	6 (1)	4.3%
*Serratia marcescens*	5 (2)	1.8%	4 (2)	2.8%
*Providencia stuartii*	4 (3)	1.5%	2 (2)	1.4%
*Citrobacter* species	4 (0)	1.5%	1 (0)	0.7%
Other Gram−	2 (0)	0.7%	2 (0)	1.4%

Abbreviations: ETA, endotracheal aspirate; BAS, bronchial aspirate; BAL, bronchoalveolar lavage; MSSA, methicillin-sensitive *S. aureus*; MRSA, methicillin-resistant *S. aureus*.

^a^1 *Enterococcus faecium* and 1 *Enterococcus faecalis*.

^b^1 *E. faecium* and 1 *E. faecalis*.

^c^In the derivation cohort, polymicrobial growth was observed in 74 of 275 patients with positive respiratory cultures, corresponding to 26.9% of cases. On the other hand, in the validation cohort, polymicrobial growth was identified in 39 of 141 patients (27.6%).

To identify independent risk factors associated with MDR infection, variables previously reported in the literature were first assessed by univariate analysis. Comparison between patients with MDR and non-MDR pneumonia identified several significant risk factors (Table 3).

**Table 3.** Risk Factors for MDR Infection in the Derivation and Validation Cohorts

Risk Factor For MDR	Derivation Cohort				Validation Cohort			
	Population (n = 275)	MDR (N = 83)	Non-MDR (N = 192)	*P* Value	Population (N = 141)	MDR (N = 55)	Non-MDR (N = 86)	*P* Value
Hospitalization (≥2 d) within the previous 90 d	87/275 (31.6%)	51/83 (61.4%)	36/192 (18.9%)	<.001	64/141 (45.4%)	39/55 (70.9%)	25/86 (29.1%)	<.001
Residence in a nursing home or long-term care facility	58/275 (21.1%)	38/83 (45.8%)	20/192 (10.4%)	<.001	41/141 (29.1%)	30/55 (54.5%)	11/86 (12.8%)	<.001
Intravenous therapy within the previous 30 d	85/269 (31.6%)	53/82 (64.6%)	32/187 (17.1%)	<.001	60/141 (42.6%)	41/55 (74.5%)	19/86 (22.1%)	<.001
Chemotherapy within the previous 30 d	22/268 (8.2%)	5/82 (6.1%)	17/186 (9.1%)	.48	17/141 (12.1%)	4/55 (7.3%)	13/86 (15.1%)	.19
Wound care within the previous 30 d	23/268 (8.6%)	14/82 (17.1%)	9/186 (4.8%)	0.002	14/141 (9.9%)	9/55 (16.4%)	5/86 (5.8%)	0.04
Dialysis within the previous 30 d	3/268 (1.1%)	2/82 (2.4%)	1/186 (0.5%)	.22	1/141 (0.7%)	1/55 (1.8%)	0/86 (0%)	.39
Hospital attendance within the previous 30 d	102/272 (37.5%)	35/83 (42.2%)	67/189 (35.5%)	.34	63/141 (44.7%)	23/55 (41.8%)	40/86 (46.5%)	.61
Antibiotic therapy within the previous 30 d	92/273 (33.7%)	51/83 (61.5%)	41/190 (21.6%)	<.001	59/141 (41.8%)	36/55 (65.5%)	23/86 (26.7%)	<.001
Antibiotic therapy within the previous 60 d	101/273 (37%)	58/83 (69.9%)	43/190 (22.6%)	<.001	65/141 (46.1%)	42/55 (76.4%)	23/86 (26.7%)	<.001
Aspiration pneumonia^a^	49/275 (17.8%)	23/83 (27.7%)	26/192 (13.5%)	.013	28/141 (19.9%)	14/55 (25.5%)	14/86 (16.3%)	.2
Tracheostomy	25/273 (9.2%)	14/83 (16.9%)	11/190 (5.8%)	.006	17/141 (12.1%)	10/55 (18.2%)	7/86 (8.1%)	.11
Enteral feeding	29/273 (10.6%)	23/83 (27.7%)	6/190 (3.2%)	<.001	18/141 (12.8%)	15/55 (27.3%)	3/86 (3.5%)	<.001
Drug-resistant pneumonia within the previous year	29/273 (10.6%)	18/83 (21.7%)	11/190 (5.8%)	<.001	20/141 (14.2%)	13/55 (23.6%)	7/86 (8.1%)	<.013
Hospitalization within the previous 60 d	78/272 (28.7%)	48/83 (57.8%)	30/189 (15.9%)	<.001	58/141 (41.1%)	38/55 (69.1%)	20/86 (23.3%)	<.001
Anti-H2 receptor antagonist or proton pump inhibitor therapy within the previous 14 d	142/275 (51.6%)	59/83 (71.1%)	83/192 (43.2%)	<.001	93/141 (66%)	45/55 (81.8%)	48/86 (55.8%)	.002
MRSA colonization within the previous year	7/274 (2.6%)	5/82 (6.1%)	2/192 (1%)	.027	4/141 (2.8%)	2/55 (3.6%)	2/86 (23.3%)	.64
Home noninvasive ventilation	8/273 (2.9%)	5/83 (6%)	3/190 (1.6%)	.041	6/140 (4.3%)	1/55 (1.8%)	0/86 (0%)	.38
Noninvasive or invasive mechanical ventilation within the previous 3 m	15/273 (5.5%)	14/83 (16.9%)	1/190 (0.5%)	<.001	12/141 (8.5%)	11/55 (20%)	1/86 (1.2%)	<.001
Positive rectal swab for MDR organisms	55/273 (20.1%)	37/83 (44.6%)	18/190 (9.5%)	<.001	39/141 (27.7%)	27/55 (49.1%)	12/86 (14%)	<.001
Poor functional status^b^	77/275 (28%)	47/83 (56.6%)	30/192 (15.6%)	<.001	54/141 (38.3%)	35/55 (63.6%)	19/86 (22.1%)	<.001
History of prior pneumonia^c^	113/275 (41.1%)	55/83 (66.3%)	58/192 (30.2%)	<.001	73/141 (51.8%)	39/55 (70.9%)	34/86 (39.5%)	<.001

^a^History of aspiration pneumonia: clinical diagnosis based on suspected or documented aspiration, in the presence of compatible clinical features and radiological evidence of pneumonia.

^b^Poor functional status defined as Karnofsky performance status < 70 or RANKIN > 2.

^c^Pneumonia occurring within the previous 2 years.

### RESPIRE Score

Variables identified in the multivariable analysis of the derivation cohort (*P* < .20) were entered into a binary logistic regression model, with MDR pneumonia as the dependent outcome. Based on this model, the RESPIRE score was developed (Table 4) (Supplementary Table 2—Supplementary Material).

**Table 4:** RESPIRE Score

Variables	Points
Hospitalization ≥2 d within the previous 90 d	1.5
Antibiotic therapy within the previous 60 d	1.5
Enteral feeding (NGT or PEG)	1
Poor functional status^a^	1
Residence in a nursing home or long-term care facility	1
Total	6
*Positive score defined as ≥2*	

Abbreviations: NGT, nasogastric tube; PEG, percutaneous endoscopic gastrostomy.

^a^Karnofsky < 70 o RANKIN > 2.

Regression coefficients were normalized and incorporated into a simplified scoring system assigning 1.5 points each for recent hospitalization and recent antibiotic use and 1 point each for enteral feeding, poor functional status (defined as Karnofsky performance status < 70 or modified Rankin score > 2), and residence in long-term care facilities.

The optimal cutoff identified using the Youden index was a total score ≥ 2, corresponding to a high risk of MDR infection (Table 4).

The performance of the RESPIRE score was evaluated in comparison with established prediction tools (Table 5).

**Table 5.** Comparison of Clinical Prediction Scores in the Derivation and Validation Cohorts

Derivation Cohort								
Prediction Model	Cutoff score	Sensitivity (95% CI)	Specificity (95% CI)	PPV (95% CI)	NPV (95% CI)	AUC (95% CI)	+LR (95% CI)	−LR (95% CI)
RESPIRE	2	0.78 (0.70–0.85)	0.78 (0.75–0.82)	0.61 (0.55–0.67)	0.89 (0.85–0.93)	0.85 (0.80–0.91)	3.63 (2.74–4.62)	0.28 (0.18–0.41)
HCAP	1	0.87 (0.78–0.93)	0.51 (0.47–0.54)	0.44 (0.40–0.47)	0.9 (0.83–0.94)	0.69 (0.62–0.75)	1.77 (1.48–2)	0.26 (0.14–0.46)
DRIP	4	0.78 (0.69–0.85)	0.76 (0.73–0.8)	0.60 (0.53–0.65)	0.89 (0.84–0.93)	0.83 (0.77–0.9)	3.32 (2.52–4.21)	0.29 (0.19–0.42)
Schreiber	2	0.46 (0.37–0.54)	0.81 (0.77–0.85)	0.51 (0.41–0.61)	0.77 (0.74–0.81)	0.73 (0.67–0.8)	2.41 (1.62–3.56)	0.67 (0.54–0.82)
Shorr	2	0.83 (0.75–0.9)	0.67 (0.63–0.7)	0.53 (0.47–0.57)	0.90 (0.85–0.94)	0.82 (0.76–0.87)	2.52 (2.03–3)	0.25 (0.15–0.4)
Aliberti	3	0.85 (0.77–0.92)	0.66 (0.62–0.69)	0.53 (0.47–0.57)	0.91 (0.86–0.95)	0.77 (0.67–0.8)	2.51 (2.03–2.93)	0.22 (0.12–0.37)
Shindo	3	0.66 (0.57–0.74)	0.81 (0.77–0.84)	0.60 (0.52–0.67)	0.84 (0.8–0.88)	0.83 (0.77–0.88)	3.40 (2.43–4.67)	0.43 (0.31–0.56)
Park	3	0.37 (0.3–0.41)	0.96 (0.93–0.98)	0.81 (0.66–0.91)	0.78 (0.75–0.79)	0.82 (0.76–0.87)	9.72 (4.35–23.56)	0.66 (0.6–0.76)

Validation Cohort								
Prediction model	Cutoff score	Sensitivity (95% CI)	Specificity (95% CI)	PPV (95% CI)	NPV (95% CI)	AUC (95% CI)	+LR (95% CI)	−LR (95% CI)
RESPIRE	2	0.89 (0.79–0.95)	0.72 (0.66–0.76)	0.67 (0.6–0.72)	0.91 (0.83–0.96)	0.88 (0.82–0.94)	3.19 (2.32–3.97)	0.15 (0.06–0.31)
HCAP	1	0.95 (0.86–0.97)	0.36 (0.3–0.37)	0.49 (0.44–0.51)	0.91 (0.77–0.98)	0.65 (0.56–0.74)	1.48 (1.23–1.61)	0.15 (0.04–0.47)
DRIP	4	0.86 (0.75–0.93)	0.74 (0.67–0.78)	0.68 (0.60–0.74)	0.89 (0.81–0.94)	0.85 (0.78–0.91)	3.26 (2.3–4.29)	0.2 (0.1–0.37)
Schreiber	2	0.51 (0.4–0.61)	0.71 (0.64–0.78)	0.53 (0.42–0.64)	0.69 (0.73–0.76)	0.69 (0.61–0.78)	1.75 (1.11–2.73)	0.69 (0.5–0.94)
Shorr	2	0.95 (0.86–0.99)	0.52 (0.46–0.54)	0.56 (0.51–0.58)	0.94 (0.83–0.98)	0.82 (0.75–0.89)	1.96 (1.58–2.16)	0.11 (0.03–0.31)
Aliberti	3	0.93 (0.83–0.98)	0.55 (0.49–0.58)	0.57 (0.51–0.57)	0.92 (0.82–0.97)	0.78 (0.7–0.85)	2.05 (1.62–2.31)	0.13 (0.04–0.34)
Shindo	3	0.73 (0.62–0.82)	0.72 (0.66–0.79)	0.64 (0.54–0.71)	0.81 (0.73–0.87)	0.83 (0.76–0.9)	2.72 (1.84–3.9)	0.37 (0.23–0.57)
Park	3	0.44 (0.35–0.49)	0.95 (0.9–0.98)	0.86 (0.69–0.95)	0.73 (0.68–0.75)	0.81 (0.74–0.88)	9.38 (3.4–30.97)	0.59 (0.52–0.73)

Abbreviations: 95% CI, 95% confidence interval; AUC, area under the receiver operating characteristic curve; LR+, positive likelihood ratio; LR−, negative likelihood ratio; NPV, negative predictive value; PPV, positive predictive value.

**Figure 1.**
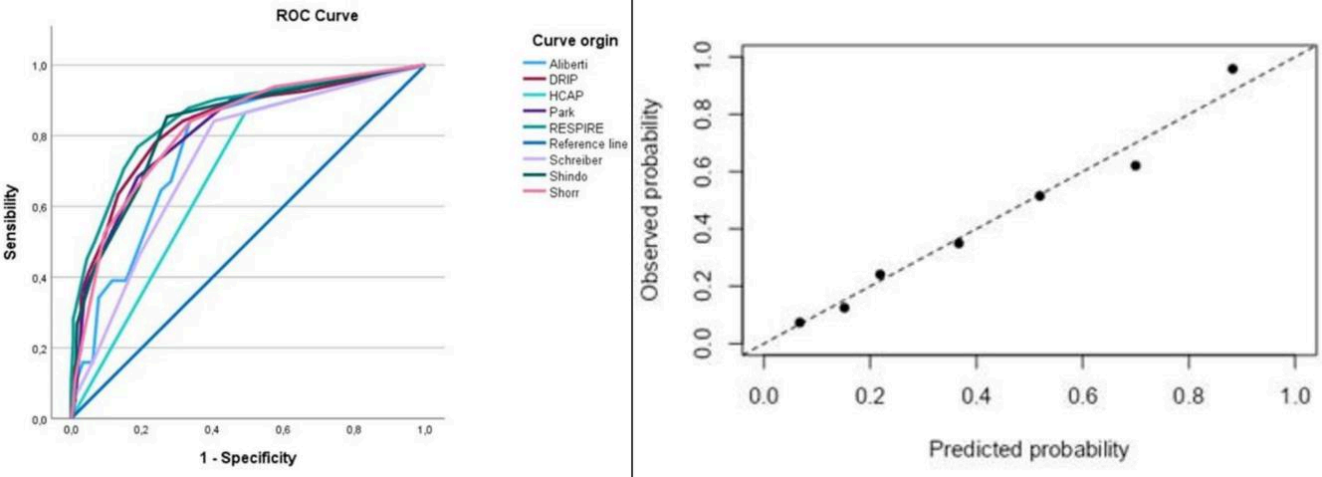
Left panel—receiver operating characteristic (ROC) curve for the derivation cohort. Right panel—calibration plot of the RESPIRE score in the derivation cohort. In the derivation cohort, the RESPIRE score demonstrated good internal calibration, as confirmed by the calibration plot and the Hosmer–Lemeshow goodness-of-fit test (χ^2^ = 4.52, df = 8; *P* = .81).

In the derivation cohort, the RESPIRE score demonstrated excellent discriminative ability, with an area under the receiver operating characteristic curve (AUC) of 0.85 (95% CI 0.80–0.91). Its performance was superior to that of all other evaluated scores, including DRIP (AUC 0.83), and markedly better than the ATS/IDSA HCAP criteria (AUC 0.69) (Table 5; Supplementary Table 3; Figure 1).

Statistical comparison of ROC curves confirmed that RESPIRE was significantly more accurate than the other scoring systems (*P* < .05), except for DRIP (*P* = .052) (Supplementary Table 4, Supplementary material).

**Figure 2.**
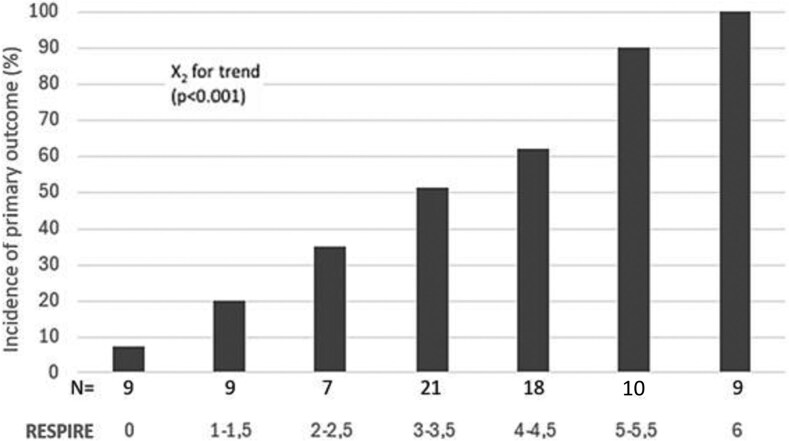
Incidence of the primary outcome according to increasing RESPIRE score categories in the derivation cohort. N = number of patients with MDR infection within each risk group.

Increasing RESPIRE score values were associated with a progressive rise in the prevalence of MDR infection across score categories (*P* < .001) (Figure 2).

Calibration of the RESPIRE score for prediction of MDR pneumonia was assessed in the derivation cohort using a calibration plot. The model demonstrated good internal calibration (Figure 1). The calibration intercept was 0.00 (standard error 0.18; 95% CI −0.36 to 0.36), and the calibration slope was 1.00 (standard error 0.12; 95% CI 0.77–1.24).

Penalized logistic regression using the least absolute shrinkage and selection operator (LASSO) confirmed the robustness of the variable selection process underlying the RESPIRE score, reducing the risk of overfitting in the derivation cohort (Supplementary Table 5).

Internal validation using bootstrap resampling showed excellent model discrimination (apparent AUC 0.85), with negligible mean optimism and an optimism-corrected AUC of 0.84, suggesting minimal overfitting and stable predictive performance (Supplementary Table 4, Supplementary material).

In the derivation cohort, application of the RESPIRE score would have resulted in undertreatment in 18/273 patients (6.6%) and overtreatment in 41/273 patients (15.0%), compared with 15/273 (5.5%) and 71/273 (26.0%) patients, respectively, using HCAP-based criteria.

### Validation Cohort

External validation of the RESPIRE score was performed in an independent cohort of 141 patients enrolled between 1 January 2024 and 31 December 2025. In this cohort, the prevalence of MDR pneumonia was 39% (55/141) (Supplementary Figure 1, study flowchart).

Baseline demographic characteristics were comparable to those of the derivation cohort, with a mean age of 72.3 ± 15.6 years and no statistically significant age differences between MDR and non-MDR subgroups (Table 1). Female patients accounted for 59/141 (41.8%) and were evenly distributed between the 2 groups.

Regarding clinical outcomes, 128/141 patients (90.1%) were hospitalized after Emergency Department presentation; 6/141 (4.3%) were discharged home; 6/141 (4.3%) were discharged to a healthcare facility; and 1/141 (0.7%) died in the Emergency Department. Overall 30-day mortality was 23.4% (33/141).

Risk factor analysis in the validation cohort confirmed the robustness of the variables included in the RESPIRE score: recent hospitalization, residence in long-term care facilities, prior antibiotic therapy, and enteral feeding remained strongly associated with MDR infection (all *P* < .001) (Table 3).

**Figure 3.**
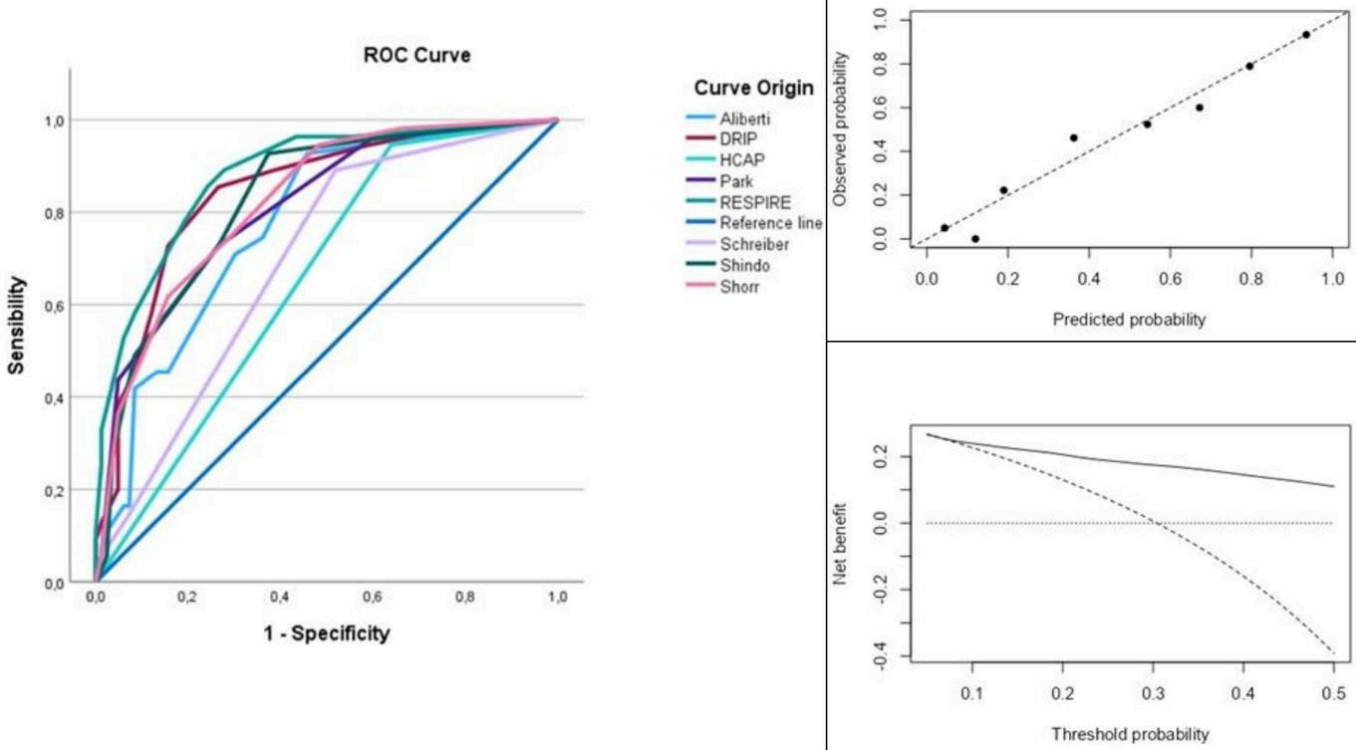
Left panel—ROC curve for the validation cohort. Right upper panel—Calibration plot of the RESPIRE score in the validation cohort. The diagonal line (y = x) represents ideal calibration. Right lower panel—decision curve analysis of the RESPIRE score in the validation cohort. The graph illustrates the net benefit of the RESPIRE model (thick black line) across a range of threshold probabilities, compared with the alternative strategies of “treat all” (dashed line) and “treat none” (thin black line).

The RESPIRE score maintained excellent discriminative performance in the validation cohort, with an AUC of 0.88 (95% CI 0.74–0.89), sensitivity of 89%, and specificity of 72% (Table 5; Figure 3).

Direct comparison with other risk stratification systems demonstrated the superiority of the RESPIRE score over all existing clinical prediction models (Supplementary Table 6, Supplementary material).

In terms of discriminative performance, RESPIRE showed a significantly higher AUC than all other evaluated systems. In particular, compared with the ATS/IDSA HCAP criteria (AUC 0.65), which maintained high sensitivity (95%) but poor specificity (36%), RESPIRE achieved markedly better overall accuracy.

Compared with the DRIP score, RESPIRE demonstrated a higher AUC in the derivation cohort (0.88 vs 0.85; *P* = .026) and greater sensitivity (89% vs 86%). The remaining scores (Schreiber, Shorr, Aliberti, Shindo, and Park) showed substantially lower performance (*P* < .01) (Table 5; Supplementary Table 6, Supplementary material).

Calibration of the RESPIRE score was assessed in the validation cohort using a calibration plot (Figure 3; Supplementary Table 7, Supplementary material). The model demonstrated good calibration, as confirmed by the Hosmer–Lemeshow goodness-of-fit test (χ^2^ = 5.67; *P* = .68). The calibration intercept was 0.00 (standard error 0.23; 95% CI −0.46 to 0.46), and the calibration slope was 1.00 (standard error 0.16; 95% CI 0.68–1.32).

Clinical utility was further evaluated using decision curve analysis (DCA), comparing the net benefit of the RESPIRE score (Figure 3). The DCA demonstrated that use of the RESPIRE score was associated with a higher net benefit across a clinically relevant range of threshold probabilities, particularly at intermediate thresholds; at higher threshold probabilities, net benefit decreased (Figure 3).

Similarly, in the validation cohort, application of the RESPIRE score would have resulted in undertreatment in 6/141 patients (4.3%) and overtreatment in 24/141 patients (17.0%), compared with 3/141 (2.1%) and 52/141 (36.9%) patients, respectively, using HCAP-based criteria.

## DISCUSSION

This study led to the derivation and validation of a novel predictive tool for identifying patients with pneumonia at risk of MDR infection.

The analysis included a derivation cohort of 275 patients and an independent validation cohort of 141 patients. The prevalence of MDR infection was substantial in both groups (30.1% in the derivation cohort and 39% in the validation cohort). Compared with international data, the MDR rates observed at our center fall within the intermediate-to-high range, likely reflecting local epidemiology and the characteristics of patients presenting to our Emergency Department. The smaller sample size observed in the prospective cohort, despite a similar study duration, reflects differences in patient selection criteria.

The high prevalence of MDR pathogens observed in our cohorts (30%–39%) should be interpreted in light of the study design, as the majority of patients underwent BAL testing. This likely selected a clinically more severe and microbiologically investigated subset of CAP patients, potentially characterized by a higher baseline risk of MDR infection compared with unselected CAP populations reported in the literature, where MDR prevalence is generally lower [27].

The final model is based on 5 clinical variables that are readily available at Emergency Department presentation during initial history taking. These risk factors are well established in the literature; however, the main strength of the proposed score lies in its simplicity and limited number of variables, distinguishing it from more complex clinical prediction tools [20, 28].

The principal finding of our study is that the RESPIRE score demonstrates excellent discriminative ability in distinguishing MDR from non-MDR-CAP. In the cohort, the score achieved an AUC of 0.88, outperforming all comparator scores.

Our findings further confirm the well-established limitations of the HCAP concept. Although the HCAP criteria demonstrated excellent sensitivity (95%), their specificity was unacceptably low (36%). This imbalance translates into substantial overtreatment in clinical practice [6]. These results reinforce that a “broad-spectrum-for-all-at-risk” approach based on overly inclusive definitions is no longer sustainable in the era of antimicrobial stewardship.

The principal clinical implication of our findings relates to therapeutic decision-making aligned with antimicrobial stewardship principles. The availability of a validated score may facilitate early de-escalation and appropriate antibiotic targeting [29]. In an exploratory analysis including patients with culture-negative and viral CAP classified as non-MDR, the RESPIRE score maintained its ability to reduce potential overtreatment compared with clinical criteria-based approaches, supporting its role in antimicrobial stewardship.

The RESPIRE score showed a strong negative predictive value in our study population, supporting its potential role as a rule-out tool for MDR pathogens, although this should be confirmed in unselected CAP populations. With a cutoff < 2, the RESPIRE score achieved a negative predictive value (NPV) of 91%, potentially supporting safe therapeutic de-escalation. Its high sensitivity, low negative likelihood ratio, and high negative predictive value support its use in identifying patients at low risk of MDR pathogens, in whom broad-spectrum antibiotic therapy may be safely avoided. However, this interpretation should be limited to populations similar to those included in the present study, and the score should not be considered a universal decision tool for all patients with suspected CAP. In patients with higher scores, treatment decisions should be guided by integration with clinical severity and individual patient factors.

Beyond discrimination, calibration is essential for the clinical applicability of a predictive model. In the validation cohort, the RESPIRE score demonstrated good calibration (Figure 3). However, the RESPIRE score was developed to support therapeutic decision-making alongside, rather than replacing, clinical judgment, which should always take into account disease severity and the overall clinical context.

Overall, the model's good calibration and the results of decision curve analysis indicate that the RESPIRE score not only effectively discriminates patients at different risks of MDR infection but also generates reliable and clinically actionable risk estimates, supporting therapeutic decisions aligned with antimicrobial stewardship principles. Reducing unnecessary broad-spectrum exposure may also contribute to limiting further selection of resistance.

Although the model showed strong discrimination, its performance should be interpreted in the context of the selected, microbiologically investigated population, and its impact on clinical outcomes and antibiotic consumption warrants prospective evaluation.

## STRENGTHS AND LIMITATIONS

Among the main strengths of this study is the detailed characterization of an Italian cohort, addressing a gap in the literature largely dominated by extra-European studies. Furthermore, all included patients had a microbiologically confirmed diagnosis based on respiratory culture results, considered the gold standard for etiologic diagnosis of pneumonia.

However, several limitations should be acknowledged. First, this was a single-center study conducted in a setting with specific local epidemiology of MDR pathogens. Although internal validation was robust, the performance of the RESPIRE score may be influenced by regional variations in pathogen distribution. Therefore, external multicenter validation in different geographical and epidemiological settings is warranted to confirm its generalizability.

Second, the retrospective and observational components of data collection may have introduced selection bias. Nevertheless, the variables included in the final model are routinely available in clinical practice.

Although respiratory samples were predominantly obtained through bronchoalveolar lavage, which reduces the risk of contamination, the possibility of colonization cannot be entirely excluded, particularly for Gram-negative organisms. However, microbiological findings were interpreted in conjunction with clinical and radiological criteria for pneumonia, which likely mitigates this limitation.

Only patients with microbiologically documented CAP were included, which may limit generalizability to unselected ED populations and introduce potential selection bias. However, this approach also represents a methodological strength, as it ensures a gold-standard etiological definition.

## CONCLUSIONS

The RESPIRE score demonstrated superior performance compared with traditional HCAP criteria and most existing clinical prediction tools for identifying MDR infection in community-acquired pneumonia, with consistent results in both derivation and validation cohorts.

Its implementation in Emergency Department protocols may improve antibiotic appropriateness by reducing unnecessary broad-spectrum use, in line with antimicrobial stewardship principles.

Prospective multicenter studies are needed to confirm generalizability and assess its impact on clinical outcomes and antibiotic consumption.

## Supplementary Material

ofag319_Supplementary_Data

## Supplementary Data

Supplementary materials are available at *Open Forum Infectious Diseases* online. Consisting of data provided by the authors to benefit the reader, the posted materials are not copyedited and are the sole responsibility of the authors, so questions or comments should be addressed to the corresponding author.
