# Supplementary material for: RESPIRE Score: Derivation and Validation of a New Risk Score for Prediction of Community-acquired Pneumonia Caused by Resistant Pathogens
Source: Open Forum Infect Dis. 2026 May 21;13(6):ofag319. doi: 10.1093/ofid/ofag319 (PMC13229114; doi:10.1093/ofid/ofag319)
Supplement: ofag319_Supplementary_Data [file ofag319_supplementary_data.doc]

MATHERIAL AND METHODS

**Diagnostic Tests for Pathogen Identification**

**Multiplex PCR (BIOFIRE® FILMARRAY® Pneumonia plus Panel)**

In routine clinical practice, patients undergoing molecular analysis had respiratory specimens collected through bronchoalveolar lavage (BAL), bronchial aspirate (BAS), or endotracheal aspirate (ETA). These samples were analyzed using syndromic molecular panel specific for community-acquired pneumonia (CAP) and healthcare-associated pneumonia (HCAP), the BIOFIRE® FILMARRAY® Pneumonia Plus Panel. The BIOFIRE® FILMARRAY® Pneumonia Plus Panel is performed on the BIOFIRE® FILMARRAY® System, it’s a multiplex PCR platform certified by the FDA, CE-IVD, and TGA. and detects 18 bacterial targets (11 Gram-negative, 4 Gram-positive, and 3 atypical pathogens), 7 antimicrobial resistance markers, and 9 viral targets associated with pneumonia and other lower respiratory tract infections (Table S1).

| **Bacteria (Semi-Quantitative)** | **Atypical Bacteria (Qualitative)** | **Viruses** | **Antimicrobial Resistance Genes** |
| --- | --- | --- | --- |
| *Acinetobacter calcoaceticus-baumannii complex*  *Enterobacter cloacae*  *Escherichia coli*  *Haemophilus influenzae*  *Klebsiella aerogenes*  *Klebsiella oxytoca*  *Klebsiella pneumoniae group*  *Moraxella catarrhalis*  *Proteus spp.*  *Pseudomonas aeruginosa*  *Serratia marcescens*  *Staphylococcus aureus*  *Streptococcus agalactiae*  *Streptococcus pn.*  *Streptococcus pyogenes* | *Legionella pneumophila*  *Mycoplasma pneumoniae*  *Chlamydia pneumoniae* | *Influenza A*  *Influenza B*  *Adenovirus*  *Coronavirus (non SARS-coV)*  *Parainfluenza virus*  *Respiratory Syncytial Virus*  *Human Rhinovirus/Enterovirus*  *Human Metapneumovirus*  *MERS-CoV* | *CTX-M*  *KPC*  *NDM*  *Oxa48-like*  *VIM*  *IMP*  *mecA/C and MREJ* |

Table S1 – Pathogens and resistance genes detected by BIOFIRE® FILMARRAY® Pneumonia plus Panel.

The overall sensitivity and specificity for BAL samples are 96.2% and 98.3%, respectively; for sputum samples, sensitivity and specificity are 96.3% and 97.2%, respectively.

The system integrates sample preparation, nucleic acid extraction and purification, amplification, detection, and analysis within a single platform. It requires approximately 2 minutes of hands-on time, with a total turnaround time of about 1 hour.

Results were communicated by telephone to the treating physicians. The Microbiology Department did not provide therapeutic recommendations.

Conventional culture testing (gold standard) was performed in parallel on all respiratory samples. When awaiting molecular panel results could potentially delay antibiotic initiation, patients received empirical antimicrobial therapy, which was subsequently adjusted according to the molecular findings once available.

No additional diagnostic procedures beyond standard clinical practice were introduced. The diagnostic and therapeutic workflow described reflects routine management of patients receiving targeted antimicrobial therapy based on multiplex PCR results.

**Culture Examination**

Lower respiratory tract (LRT) samples were processed using conventional culture methods for the detection of bacterial and fungal pathogens. Ten microliters of respiratory samples fluid were inoculated onto non-selective media, including blood agar, CHROMagar^TM^ Candida incubated at 37° and chocolate agar , incubated at 37 °C in a 5% CO₂ atmosphere. Microbial growth was assessed after 36 and 48 hours of incubation.

Bacterial colonies were identified by matrix-assisted laser desorption/ionization time-of-flight mass spectrometry (MALDI-TOF MS- MALDI Biotyper®, Bruker, US). Potential pathogens were reported semi-quantitatively as colony-forming units per milliliter (CFU/mL). Antimicrobial susceptibility testing (AST) was performed using automated systems such as BD Phoenix (Bruker US), Vitek®2 (bioMérieux) or commercial broth microdilution (Merlin Diagnostika GmbH, Germany).

.

**Index Tests for Fungal Pathogen Identification**

**Conventional Culture**

LRT samples, including bronchoalveolar lavage (BAL), were processed using standard culture techniques as described above for bacterial and fungal detection.

**Quantitative PCR for *Aspergillus spp.*, *Pneumocystis jirovecii*. (Respiratory Samples)**

Quantitative PCR assays were performed on respiratory specimens (BAL, tracheal aspirate, or sputum) to detect DNA from *Aspergillus spp.* and *Pneumocystis jirovecii*. . These molecular assays were performed by automated platform singleplex RT-PCR (Elite MGB® Kit Elite Ingenius- ElithechGroup).

These tests have high sensitivity with rapid turnaround time andrepresent a valuable tool for early diagnosis, particularly in critically ill or immunocompromised patients.

**Serum/Plasma/respiratory sample Galactomannan and serum β-D-Glucan**

Galactomannan test in serum and respiratory samples assay was performed by chemiluminescent immunoassay (Monotest Virclia- Vircell SL) and serum β-D-glucan levels were measured using a quantitative chromogenic method (Fungus (1-3)-β- Glucan Test—Genobio/Era Biology). .

Galactomannan, a component of the cell wall of *Aspergillus spp.*, is released into the bloodstream during invasive growth. β-D-glucan is a polysaccharide present in the cell wall of several fungi, including *Candida*, *Aspergillus*, *Fusarium*, and *Pneumocystis jirovecii*, but not *Cryptococcus* spp. or Mucorales.

Detection of these circulating antigens may support the diagnosis of invasive fungal infection; however, results must be interpreted in conjunction with the patient’s clinical presentation, radiological findings, and microbiological data.

**Additional statical analysis**

To assess the added value of the RESPIRE score beyond known high-risk features, we performed a secondary analysis excluding patients with prior MDR isolation or colonization, as these individuals would typically receive empirical broad-spectrum therapy irrespective of prediction tools. In this restricted population, the performance of the RESPIRE score was re-evaluated in terms of discrimination (AUROC), sensitivity, specificity, predictive values, and likelihood ratios.

To assess the robustness of the model, we performed an additional exploratory sensitivity analysis using a conservative worst-case scenario approach, in which all patients with culture-negative results were classified as non-MDR. The performance of the RESPIRE score was then re-evaluated in this expanded population in terms of discrimination (AUC), sensitivity, specificity, predictive values, and likelihood ratios. Detailed results of this analysis are reported in the Supplementary Material.

RESULTS


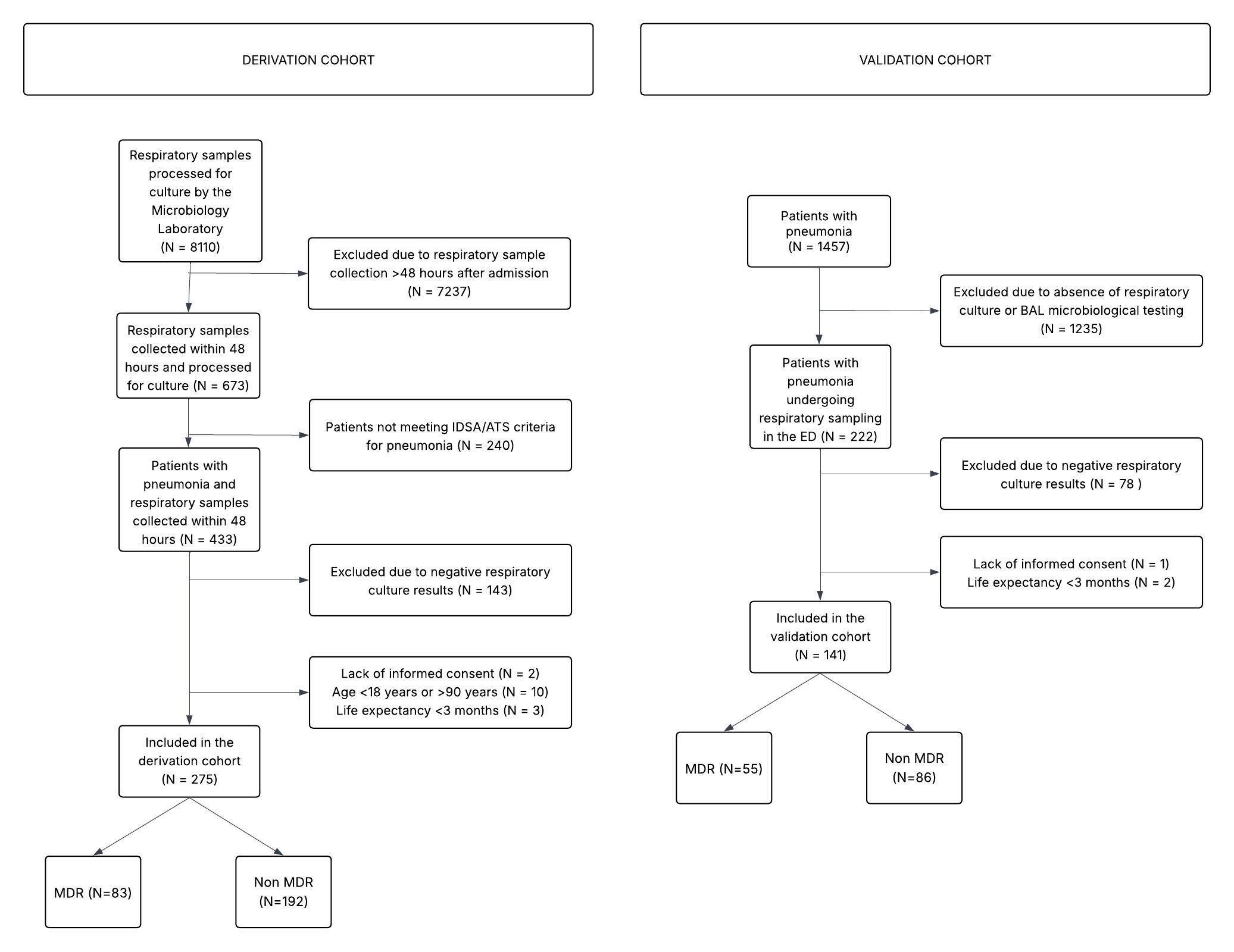


Figure S1 - Study flow chart showing the patients workup and the steps from the initial assessment of eligibility to the final analysis. Left: derivation cohort. Right: validation cohort.

| VARIABLES | DERIVATION COHORT | | | | VALIDATION COHORT | | | |
| --- | --- | --- | --- | --- | --- | --- | --- | --- |
|  | **Population (n=275)** | **MDR (N=83)** | **Non-MDR (N=192)** | **P value** | **Population (N=141)** | **MDR (N=55)** | **Non-MDR (N=86)** | **P value** |
| Vitals |  | | | | | | | |
| HR (bpm) | 94.4 ± 21.8 | 94.9 ± 24.6 | 94.2 ± 20.5 | 0.06 | 94.3 ± 19.3 | 94.3 ± 21.7 | 94.2 ± 17.8 | 0.08 |
| SBP (mmHg) | 121.7 ± 27.7 | 122.5 ± 26.2 | 121.3 ± 28.4 | 0.90 | 120.8 ± 24.2 | 123.0 ± 23.0 | 119.3 ± 24.9 | 0.71 |
| DBP (mmHg) | 71.4 ± 15.9 | 70.6 ± 13.8 | 71.8 ± 16.8 | 0.10 | 69.9 ± 13.5 | 70.2 ± 13.1 | 69.7 ± 13.8 | 0.49 |
| SpO2 | 92.3 ± 8 | 93.1 ± 6.8 | 92.0 ± 8.5 | 0.24 | 92.1 ± 7.9 | 93.1 ± 6.8 | 91.5 ± 8.6 | 0.25 |
| FiO2 % | 38.32 ± 23.9 | 37.8 ± 20.2 | 38.6 ± 25.4 | 0.07 | 35 ± 18.2 | 35.5 ± 16.2 | 34.7 ± 19.4 | 0.5 |
| RR (rpm) | 22.6 ± 7.5 | 23.7 ± 8.2 | 22.1 ± 7.1 | 0.22 | 24 ± 7.3 | 24.2 ± 8.4 | 23.9 ± 6.6 | **0.01** |
| GCS | 12.9 ± 4 | 13.5 ± 3.4 | 12.6 ± 4.2 | **0.001** | 14.1 ± 2.4 | 14.3 ± 2.1 | 13.9 ± 2.7 | 0.13 |
| BT°C | 36.9 ± 1.1 | 37.0 ± 1.2 | 36.8 ± 1.1 | 0.33 | 37.1 ± 1.1 | 37.2 ± 1.1 | 37.1 ± 1.1 | 0.95 |
| ABG |  | | | | | | | |
| pH | 7.38 ± 0.13 | 7.41 ± 0.09 | 7.37 ± 0.15 | **0.001** | 7.42 ± 0.10 | 7.44 ± 0.07 | 7.41 ± 0.11 | 0.12 |
| pO2  mmHg | 79.6 ± 60.3 | 74.6 ± 41.5 | 81.9 ± 67.1 | 0.09 | 70.6 ± 45.7 | 67.2 ± 30.0 | 72.7 ± 53.2 | 0.31 |
| pCO2  mmHg | 41.9 ± 16.1 | 40.9 ± 10.7 | 42.3 ± 18.1 | **0.01** | 42.0 ± 17.3 | 39.0 ± 10.2 | 43.9 ± 20.3 | **0.01** |
| Lactate mmol/L | 2.6 ± 2.8 | 2.1 ± 1.9 | 2.8 ± 3.3 | **0.01** | 2.1 ± 2.3 | 2.0 ± 1.9 | 2.2 ± 2.5 | 0.36 |
| HCO3- mEq/L | 24.4 ± 6.1 | 25.6 ± 5.4 | 23.8 ± 6.3 | 0.24 | 26.2 ± 5.8 | 26.0 ± 5.5 | 26.3 ± 6.0 | 0.5 |
| Glucose mg/dL | 141.3 ± 60.0 | 134.5 ± 42.5 | 144.3 ± 66.3 | 0.07 | 140.4 ± 62.5 | 134.8 ± 38.8 | 143.8 ± 73.4 | 0.09 |
| FiO2 | 40.4 ± 25.5 | 38.7 ± 22.3 | 41.1 ± 26.8 | 0.07 | 38.3 ± 23.2 | 38.3 ± 22.5 | 38.2 ± 23.8 | 0.71 |
| P/F (HI) | 227.5 ± 103.2 | 226.0 ± 100.0 | 228.0 ± 105.0 | 0.70 | 223.6 ± 95.9 | 221.3 ± 91.7 | 225.0 ± 99.0 | 0.74 |
| Labs |  | | | | | | | |
| WBC (×10⁹/L) | 13.2 ± 8.1 | 12.8 ± 8.6 | 13.5 ± 7.9 | 0.85 | 12.9 ± 8.7 | 11.8 ± 8.9 | 13.6 ± 8.6 | 0.51 |
| Hb g/dl | 12.4 ± 2.3 | 12.5 ± 2.1 | 12.4 ± 2.4 | 0.18 | 12.1 ± 2.1 | 12.1 ± 2.1 | 12.1 ± 2.1 | 0.97 |
| Haematocrit % | 37.6 ± 6.4 | 37.9 ± 5.9 | 37.5 ± 6.7 | 0.32 | 37.0 ± 6.1 | 36.9 ± 6.2 | 37.1 ± 6.1 | 0.73 |
| PLT (×10⁹/L) | 270.6 ± 121.6 | 262.8 ± 120.2 | 274.1 ± 122.4 | 0.68 | 282.8 ± 130.1 | 260.7 ± 120.7 | 297.1 ± 134.6 | 0.25 |
| Lymphocyte (×10⁹/L) | 1.3 ± 1.3 | 1.3 ± 1.4 | 1.3 ± 1.3 | 0.81 | 1.1 ± 1.1 | 1.2 ± 1.3 | 1.1 ± 0.99 | 0.5 |
| PCR mg/L | 111.4 ± 107.4 | 116.9 ± 101.4 | 109 ± 110.1 | 0.50 | 116.2 ± 98.2 | 121.3 ± 98.4 | 113 ± 98.5 | 0.42 |
| PCT ng/L | 12.1 ± 44.0 | 4.6 ± 11.9 | 15.7 ± 52.4 | **0.01** | 10.1 ± 42.6 | 3.5 ± 10.1 | 14.5 ± 54.1 | **0.01** |
| NT-proBNP pg/mL | 8161.5 ± 15646.1 | 8553.2 ± 17233.9 | 7969.2 ± 14962.8 | 0.72 | 9852.1 ± 16554.0 | 11080.9 ± 19338.5 | 9093.0 ± 14839.7 | 0.35 |
| Creatinine mg/mL | 1.26 ± 0.95 | 1.14 ± 0.74 | 1.31 ± 1.03 | 0.14 | 1.14 ± 0.77 | 1.15 ± 0.79 | 1.13 ± 0.77 | 0.9 |
| Urea | 1.1 ± 0.6 | 1.1 ± 0.5 | 1.1 ± 0.61 | 0.41 | 1.1 ± 0.5 | 1.2 ± 0.5 | 1.0 ± 0.5 | 0.95 |
| INR | 1.2 ± 0.3 | 1.2 ± 0.4 | 1.2 ± 0.2 | **0.01** | 1.3 ± 0.4 | 1.3 ± 0.5 | 1.2 ± 0.3 | **0.01** |
| ALT U/L | 51.0 ± 92.8 | 57.7 ± 98.4 | 47.8 ± 90.3 | 0.09 | 34.3 ± 62.8 | 38.8 ± 73.2 | 31.4 ± 55.3 | 0.18 |
| Bilirubin mg/dL | 0.6 ± 0.4 | 0.6 ± 0.5 | 0.6 ± 0.4 | 0.31 | 0.6 ± 0.5 | 0.6 ± 0.5 | 0.6 ± 0.5 | 0.97 |
| Na+ mmol/L | 138.6 ± 5.9 | 138.6 ± 5.9 | 138.6 ± 5.9 | 0.78 | 138.4 ± 6.1 | 138.5 ± 6.6 | 138.4 ± 5.8 | 0.44 |
| K+ mmol/L | 4.3 ± 0.7 | 4.2 ± 0.7 | 4.3 ± 0.7 | 0.71 | 4.2 ± 0.7 | 4.2 ± 0.7 | 4.3 ± 0.7 | 0.72 |

***Table S1 -*** ***Arterial Blood Gas and Hematologic Parameters in the Derivation and Validation Cohorts****. HR – Heart Rate; SBP – Systolic Blood Pressure; DBP – Diastolic Blood Pressure; SpO₂ – Peripheral Oxygen Saturation; FiO₂ – Fraction of Inspired Oxygen; RR – Respiratory Rate; GCS – Glasgow Coma Scale; BT – Body Temperature; ABG: Arterial Blood Gas analysis; WBC (×10⁹/L) – White Blood Cell Count; Hb (g/dL) – Hemoglobin; Hct (%) – Hematocrit; PLT – Platelet Count; CRP – C-Reactive Protein; PCT – Procalcitonin; NT-proBNP – N-terminal pro–B-type Natriuretic Peptide; Creatinine – Serum Creatinine; INR – International Normalized Ratio; ALT – Alanine Aminotransferase; Total Bilirubin – Total Bilirubin; Na⁺ – Sodium; K⁺ – Potassium.*

| Variables included | Univariate analysis | | Multivariate analysis | |
| --- | --- | --- | --- | --- |
|  | Odds ratio (CI 95%) | P value | Odds ratio (CI 95%) | P value |
| Hospitalization (≥2 days) within the previous 90 days | 6.91 (3.90-12.23) | **<0.001** | **3.64 (1.69-7.83)** | **0.001** |
| Antibiotic therapy within the previous 60 days | 7.93 (4.45-14.15) | **<0.001** | **3.56 (1.66-7.61)** | **0.001** |
| Poor functional status* | 7.05 (3.93-12.63) | **<0.001** | **2.43 (1.10-5.39)** | **0.028** |
| Enteral feeding | 11.75 (4.57-30.23) | **<0.001** | **3.08 (0.98-9.65)** | **0.045** |
| Residence in a nursing home or long-term care facility | 7.26 (3.86-13.69) | **<0.001** | **2.2 (1.09-4.34)** | **0.047** |
| Non-invasive or invasive mechanical ventilation within the previous 3 months | 38.35 (4.95-297.10) | **<0.001** | **/** | **/** |
| Intravenous therapy within the previous 30 days | 8.85 (4.90-15.99) | **<0.001** | **/** | **/** |
| Positive rectal swab for MDR organisms | 7.69 (4.01-14.73) | **<0.001** | **/** | **/** |
| Hospitalization within the previous 60 days | 7.27 (4.05-13.04) | **<0.001** | **/** | **/** |
| MRSA colonization within the previous year | 6.17 (1.17-32.48) | **0.027** | **/** | **/** |
| Antibiotic therapy within the previous 30 days | 5.79 (3.30-10.15) | **<0.001** | **/** | **/** |
| History of prior pneumonia | 4.53 (2.62-7.86) | **<0.001** | **/** | **/** |
| Drug-resistant pneumonia within the previous year | 4.50 (2.01-10.05) | **<0.001** | **/** | **/** |
| Wound care within the previous 30 days | 4.09 (1.67-9.79) | **0.002** | **/** | **/** |
| Home non-invasive ventilation | 4.04 (0.94-17.31) | **0.041** | **/** | **/** |
| Tracheostomy | 3.30 (1.43-7.63) | **0.006** | **/** | **/** |
| Anti-H2 receptor antagonist or proton pump inhibitor therapy within the previous 14 day | 3.23 (1.86-5.62) | **<0.001** | **/** | **/** |
| Aspiration pneumonia | 2.45 (1.33-4.51) | **0.013** | **/** | **/** |

Table S2 - Score Derivation.

| Test Result Pairs | Asymptotic | | AUC Difference | **Standard Error of the Difference**ᵇ | Asymptotic 95% Confidence Interval | |
| --- | --- | --- | --- | --- | --- | --- |
|  | z | Two-Tailed Significance (p value)**ᵃ |  |  | Lower Limit | Upper Limit |
| RESPIRE – HCAP | 7.460 | **.000** | .166 | .227 | .123 | .210 |
| RESPIRE - DRIP | 1.947 | .052 | .025 | .230 | .000 | .050 |
| RESPIRE – Shorr | 2.091 | **.037** | .035 | .229 | .002 | .068 |
| RESPIRE – Park | 2.779 | **.005** | .039 | .229 | .011 | .066 |
| RESPIRE – Shindo | 2.589 | **.010** | .029 | .228 | .007 | .050 |
| RESPIRE – Aliberti | 3.525 | **.000** | .080 | .233 | .036 | .125 |
| RESPIRE – Schreiber | 4.703 | **.000** | .120 | .236 | .070 | .170 |
| HCAP – DRIP | -5.912 | **.000** | -.142 | .233 | -.189 | -.095 |
| HCAP – Shorr | -5.234 | **.000** | -.131 | .231 | -.181 | -.082 |
| HCAP – Park | -5.655 | **.000** | -.128 | .231 | -.172 | -.083 |
| HCAP – Shindo | -6.059 | **.000** | -.138 | .231 | -.182 | -.093 |
| HCAP – Aliberti | -3.295 | **.001** | -.086 | .235 | -.138 | -.035 |
| HCAP – Schreiber | -1.591 | .112 | -.046 | .238 | -.103 | .011 |
| DRIP – Shorr | .473 | .636 | .010 | .235 | -.032 | .053 |
| DRIP – Park | .775 | .438 | .014 | .234 | -.021 | .050 |
| DRIP – Shindo | .245 | .806 | .004 | .234 | -.028 | .035 |
| DRIP – Aliberti | 2.117 | **.034** | .055 | .239 | .004 | .107 |
| DRIP – Schreiber | 3.863 | **.000** | .095 | .242 | .047 | .144 |
| Shorr – Park | .182 | .855 | .004 | .233 | -.037 | .044 |
| Shorr – Shindo | -.335 | .738 | -.006 | .233 | -.043 | .031 |
| Shorr – Aliberti | 2.150 | **.032** | .045 | .237 | .004 | .086 |
| Shorr – Schreiber | 2.685 | **.007** | .085 | .240 | .023 | .147 |
| Park - Shindo | -.702 | .483 | -.010 | .232 | -.038 | .018 |
| Park – Aliberti | 1.727 | .084 | .041 | .237 | -.006 | .088 |
| Park – Schreiber | 4.564 | **.000** | .081 | .239 | .046 | .116 |
| Shindo – Aliberti | 2.233 | **.026** | .051 | .237 | .006 | .097 |
| Shindo – Schreiber | 3.376 | **.001** | .091 | .240 | .038 | .145 |
| Aliberti – Schreiber | 1.210 | .226 | .040 | .245 | -.025 | .105 |
| a. Null hypothesis: true difference in area = 0. | | | | | | |
| b. Based on the nonparametric assumption. | | | | | | |

***Table S3*** – Comparison of ROC Curves of the Scores in the Derivation Cohort

| Risk Group | n | Mean Predicted Risk | Observed Risk |
| --- | --- | --- | --- |
| 1 | **55** | **0.069** | **0.073** |
| 2 | **54** | **0.069** | **0.093** |
| 3 | **55** | **0.165** | **0.145** |
| 4 | **54** | **0.445** | **0.463** |
| 5 | **55** | **0.770** | **0.745** |

Table S4 - **Calibration of the RESPIRE Score in the Derivation Cohort.** The table reports, for each of the risk groups obtained by ranking predicted probabilities, the mean predicted risk, the observed risk of MDR pneumonia, and the sample size of each group.

| Predictor | LASSO Coefficient (λ₁se) | Selected (Coefficient ≠ 0) |
| --- | --- | --- |
| Hospitalization (≥2 days) within the previous 90 days | 0,544985 | TRUE |
| Antibiotic therapy within the previous 60 days | 0,538310 | TRUE |
| Poor functional status | 0,383298 | TRUE |
| Residence in a nursing home or long-term care facility | 0,420319 | TRUE |
| Enteral feeding (NGT/PEG) | 0,320522 | TRUE |

***Table S5*** *-* ***Penalized LASSO Logistic Regression (λ₁se) for Prediction of MDR Pneumonia – RESPIRE Score.*** *The reported coefficients derive from a LASSO-penalized logistic regression model at the λ₁se penalty value, selected through cross-validation. All RESPIRE score variables retained non-zero coefficients, indicating stable variable selection and a low risk of overfitting in the derivation cohort.*

| Test Result Pairs | Asymptotic | | AUC difference | **Standard Error of the Difference**ᵇ | Asymptotic 95% Confidence Interval | |
| --- | --- | --- | --- | --- | --- | --- |
|  | z | Two-Tailed Significance (p value)**ᵃ |  |  | Lower Limit | Upper Limit |
| RESPIRE – HCAP | 7.641 | **.000** | .227 | .243 | .168 | .285 |
| RESPIRE – DRIP | 2.219 | **.026** | .035 | .248 | .004 | .066 |
| RESPIRE – Shorr | 2.190 | **.029** | .057 | .250 | .006 | .108 |
| RESPIRE – Park | 3.342 | **.001** | .069 | .251 | .029 | .110 |
| RESPIRE – Shindo | 3.315 | **.001** | .054 | .250 | .022 | .086 |
| RESPIRE – Aliberti | 3.575 | **.000** | .104 | .258 | .047 | .161 |
| RESPIRE – Schreiber | 4.805 | **.000** | .187 | .266 | .111 | .263 |
| HCAP – DRIP | -5.690 | **.000** | -.192 | .253 | -.258 | -.126 |
| HCAP – Shorr | -4.746 | **.000** | -.170 | .254 | -.240 | -.100 |
| HCAP – Park | -4.662 | **.000** | -.158 | .255 | -.224 | -.091 |
| HCAP – Shindo | -5.478 | **.000** | -.173 | .254 | -.235 | -.111 |
| HCAP – Aliberti | -3.354 | **.001** | -.123 | .262 | -.194 | -.051 |
| HCAP – Schreiber | -.926 | .354 | -.040 | .270 | -.124 | .044 |
| DRIP – Shorr | .737 | .461 | .022 | .259 | -.037 | .081 |
| DRIP – Park | 1.199 | .231 | .034 | .261 | -.022 | .090 |
| DRIP – Shindo | .750 | .453 | .019 | .259 | -.030 | .068 |
| DRIP – Aliberti | 1.951 | .051 | .069 | .268 | .000 | .138 |
| DRIP – Schreiber | 3.780 | .**000** | .152 | .275 | .073 | .231 |
| Shorr - Park | .415 | .678 | .012 | .261 | -.045 | .069 |
| Shorr – Shindo | -.114 | .910 | -.003 | .261 | -.060 | .053 |
| Shorr – Aliberti | 1.581 | .114 | .047 | .268 | -.011 | .105 |
| Shorr – Schreiber | 2.806 | **.005** | .130 | .277 | .039 | .220 |
| Park - Shindo | -.662 | .508 | -.015 | .261 | -.061 | .030 |
| Park - Aliberti | 1.209 | .227 | .035 | .269 | -.022 | .091 |
| Park – Schreiber | 4.215 | **.000** | .118 | .276 | .063 | .172 |
| Shindo – Aliberti | 1.782 | .075 | .050 | .269 | -.005 | .105 |
| Shindo – Schreiber | 3.091 | **.002** | .133 | .277 | .049 | .217 |
| Aliberti – Schreiber | 1.789 | .074 | .083 | .284 | -.008 | .174 |
| a. Null hypothesis: true difference in area = 0. | | | | | | |
| b. Based on the nonparametric assumption. | | | | | | |

***Table S6*** *-* Comparison of ROC Curves of the Scores in the Derivation Cohort

| Risk Group | n | Mean Predicted Risk | Observed Risk |
| --- | --- | --- | --- |
| 1 | 0,048 | 0,069 | 29 |
| 2 | 0,112 | 0,036 | 28 |
| 3 | 0,323 | 0,321 | 28 |
| 4 | 0,612 | 0,679 | 28 |
| 5 | 0,868 | 0,857 | 28 |

***Table S7*** *-* ***Calibration of the RESPIRE Score in the Validation Cohort.*** *The table reports, for each of the ten risk groups defined by ranking predicted probabilities, the mean predicted risk, the observed risk of MDR pneumonia, and the sample size of each group.*

**Additional analysis**

After excluding patients with prior MDR isolation or colonization (n=34 in the derivation cohort and n=22 in the validation cohort), the RESPIRE score maintained good discriminative performance (derivation cohort AUROC 0.85, 95% CI 0.79–0.91; validation cohort AUROC 0.88, 95% CI 0.81–0.94), supporting its utility beyond known high-risk cases.

In derivation cohort after excluding patients with prior MDR isolation or colonization, the RESPIRE score showed a sensitivity of 75%, specificity of 81%, positive predictive value of 58%, and negative predictive value of 90%. The positive and negative likelihood ratios were 3.9 and 0.31, respectively.

In validation cohort, after excluding patients with prior MDR isolation or colonization, the RESPIRE score showed a sensitivity of 85%, specificity of 77%, positive predictive value of 66%, and negative predictive value of 91%. The positive and negative likelihood ratios were 3.7 and 0.20, respectively.

Including the previously excluded patients, the overall prevalence decreased to 83/418 (19.8%), assuming a priori, in a conservative worst-case scenario, that all such cases were non-MDR.

In an exploratory sensitivity analysis, we conducted a conservative worst-case scenario by classifying all culture-negative patients as non-MDR (n=143). In this setting, the RESPIRE score maintained good discriminative performance (AUROC 0.80, 95% CI 0.74–0.85), compared with 0.68 (95% CI 0.62–0.74) for HCAP-based criteria.

The RESPIRE score showed a sensitivity of 77%, specificity of 66%, positive predictive value of 37%, and negative predictive value of 92%, with positive and negative likelihood ratios of 2.3 and 0.35, respectively. In comparison, HCAP criteria showed a sensitivity of 82%, specificity of 54%, positive predictive value of 31%, and negative predictive value of 92%, with likelihood ratios of 1.8 and 0.33, respectively.

In terms of clinical impact, application of the RESPIRE score would have resulted in undertreatment in 18/418 patients (4.5%) and overtreatment in 111/418 patients (26.5%), compared with 15/418 (3.6%) and 152/418 (36.4%) patients, respectively, using HCAP-based criteria (p<0.001 in both cases).
